# 4-{2-[(3,4-Dichloro­phen­yl)(meth­yl)amino]-4-methyl-1,3-thia­zol-5-yl}-*N*-(3-methyl­phen­yl)pyrimidin-2-amine

**DOI:** 10.1107/S1600536810053547

**Published:** 2011-01-08

**Authors:** Hai-Bo Li, Hai-Bo Shi, Wei-Xiao Hu

**Affiliations:** a Nantong Center for Disease Control and Prevention, Nantong 226007, People’s Republic of China; b Zhejiang Pharmaceutical College, Ningbo 315100, People’s Republic of China; c College of Pharmaceutical Science, Zhejiang University of Technology, Hangzhou 310032, People’s Republic of China

## Abstract

In the title compound, C_22_H_19_Cl_2_N_5_S, the thia­zole and pyrimidine rings are almost co-planar, making a dihedral angle of 6.48 (7)°. In the crystal, intermolecular N—H⋯N hydrogen bonds link pairs of molecules into centrosymmetric dimers..

## Related literature

For general background to the biological activity of thia­zole derivatives, see: Narayana *et al.* (2004 ▶). For the synthesis of the title compound, see: Bredereck *et al.* (1964 ▶).
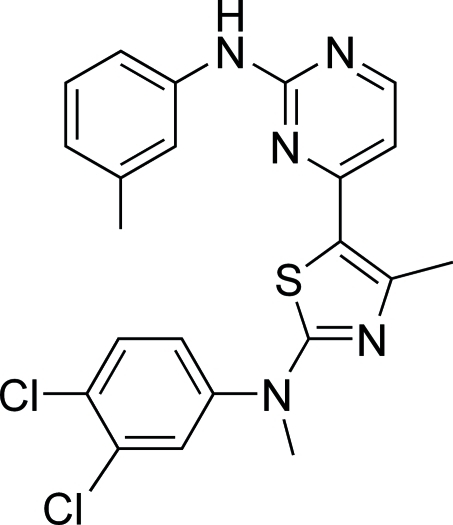


## Experimental

### 

#### Crystal data

C_22_H_19_Cl_2_N_5_S
*M* *_r_* = 456.38
Triclinic, 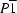

*a* = 7.9679 (15) Å
*b* = 9.4042 (19) Å
*c* = 14.166 (3) Å
α = 85.421 (6)°
β = 76.727 (5)°
γ = 86.002 (6)°
*V* = 1028.4 (3) Å^3^
*Z* = 2
Mo *K*α radiation
μ = 0.44 mm^−1^
*T* = 103 K
0.53 × 0.50 × 0.40 mm

#### Data collection

Rigaku AFC10/Saturn724+ diffractometer
Absorption correction: multi-scan (*CrystalClear*; Rigaku/MSC, 2008) ▶ *T*_min_ = 0.801, *T*_max_ = 0.844
9828 measured reflections
4626 independent reflections
3906 reflections with *I* > 2σ(*I*)
*R*_int_ = 0.021

#### Refinement

*R*[*F* ^2^ > 2σ(*F* ^2^)] = 0.033
*wR*(*F* ^2^) = 0.091
*S* = 1.05
4626 reflections
275 parameters
H-atom parameters constrained
Δρ_max_ = 0.37 e Å^−3^
Δρ_min_ = −0.25 e Å^−3^

### 

Data collection: *CrystalClear* (Rigaku/MSC, 2008 ▶); cell refinement: *CrystalClear*; data reduction: *CrystalClear*; program(s) used to solve structure: *SHELXS97* (Sheldrick, 2008 ▶); program(s) used to refine structure: *SHELXL97* (Sheldrick, 2008 ▶); molecular graphics: *SHELXTL* (Sheldrick, 2008 ▶); software used to prepare material for publication: *publCIF* (Westrip, 2010 ▶).

## Supplementary Material

Crystal structure: contains datablocks I, global. DOI: 10.1107/S1600536810053547/ng5079sup1.cif

Structure factors: contains datablocks I. DOI: 10.1107/S1600536810053547/ng5079Isup2.hkl

Additional supplementary materials:  crystallographic information; 3D view; checkCIF report

## Figures and Tables

**Table 1:** Hydrogen-bond geometry (Å, °)

*D*—H⋯*A*	*D*—H	H⋯*A*	*D*⋯*A*	*D*—H⋯*A*
N5—H5*N*⋯N3^i^	0.88	2.20	3.078 (2)	177

Symmetry code: (i) 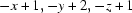.

**Fig. 1.**
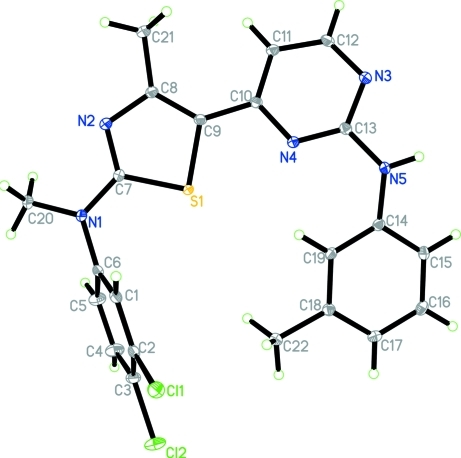
The structure of (I), shown with 30% probability displacement ellipsoids.

**Fig. 2.**
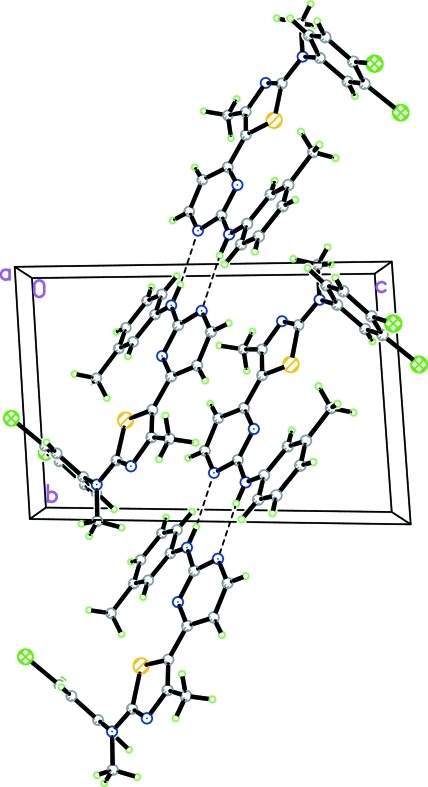
Packing of the molecules down a axis. Dashed lines denote intermolecular N—H···N hydrogen bonds.

## supplementary crystallographic information

### Comment

Thiazole derivatives are found to be associated with various biological activities (Narayana *et al.*, 2004). In order to further study the structure-activity relationship (SAR) of the thiazolyl-pyrimidine derivatives, we introduced arylamino group into 2-position of thiazole ring of thiazolyl-pyrimidine according to the general pyrimidine condensation method of Bredereck (Bredereck *et al.*, 1964). But, it was found that the obtained compound was not desired compound that confirmed by ^1^H NMR, MS. So, the structure of (I) was further determined using single-crystal X-ray diffraction.

The molecular structure of (I) is illustrated in Fig. 1. The thiazole ring (S1/C7/N2/C8/C9) and the pyrimidine ring (C10/C11/C12/N3/C13/N4) are almost planar, with a dihedral angle of 6.48 (7)°. The aniline rings (C1/C2/C3/C4/C5/C6/N1) and (C14/C15/C16/C17/C18/C19/N5) make dihedral angles of 73.76 (8) Å and 14.16 (7) Å with the thiazole ring, respectively. In the thiazole ring, the bond lengths S1—C7 [1.739 (15) Å], S1—C9 [1.749 (15) Å] and N2—C8 [1.377 (19) Å] correspond to typical single bond, and the C7—N2 [1.310 (2) Å], C8—C9 [1.372 (2) Å] belong to typical for double bonds. The crystal structure is stabilized by intermolecular weak N—H···N interactions (Fig. 2).  Furthermore, every two molecules containing two  N—H···N hydrogen bondings consists a dimer as octagon.

### Experimental

A mixture of

3-dimethylamino-1-{2-[(3,4-Dichloro-phenyl)-methyl-amino]-4-methyl-thiazol-5-yl}-propenone (1.85 g, 5 mmol) and NaOH (0.2 g, 5 mmol) in 2-methoxylethanol (40 ml) was treated with *N*-*m*-tolyl-guanidine carbonate (1.58 g, 7.5 mmol). The reaction mixture was heated at 383 K under N_2_ for 21 h. After concentration, the residue was filtered and washed liberally with ethanol and water. Recrystallization from THF affored the title compound as dark-brown crystals, 0.34 g, m.p.472–473 K, yield 15.0%. Since the crystal product was not found to be suitable for X-ray diffraction studies, a few crystals were dissolved in 2-butanone, which was allowed to evaporate slowly to give yellow crystals of (I) suitable for X-ray diffraction studies. ^1^H NMR(CDCl_3_, TMS, 400 MHz, δp.p.m.): 8.30 (d, 1H, *J* =5.2 Hz, py—H), 7.61 (s, 1H, Ar—H), 7.57 (d, 1H, *J* = 2.8 Hz, Ar—H), 7.51 (d, 1H, *J* = 8.8 Hz, Ar—H), 7.33 (dd, 1H, *J*_1_= 2.4 Hz, *J*_2 _= 2.4 Hz, Ar—H), 7.23–7.15 (m, 2H, Ar—H), 7.04 (s, 1H, Ar—H), 6.84 (d, 1H, *J* = 5.6 Hz, py—H), 3.54 (s, 3H, CH_3_), 2.59 (s, 3H, CH_3_), 2.27 (s, 3H, CH_3_). EIMS m/*z* (%): 455 (*M*^+^, 100), 440 (9), 295 (11), 256 (12), 222 (13), 213 (9), 185 (10),171 (8), 129 (19), 111 (9), 97 (16), 85 (19), 73 (41), 65 (8), 57 (48).

### Refinement

All H atoms were placed in calculated positions (C—H 0.95–0.98 Å, N—H 0.87–0.89 Å) and refined as riding with *U*_iso_(H) = 1.2–1.22Ueq of the parent atom.

### Crystal data

**Table:**

C_22_H_19_Cl_2_N_5_S	*Z* = 2
*M**_r_* = 456.38	*F*(000) = 472
Triclinic, *P*1	*D*_x_ = 1.474 Mg m^−^^3^
Hall symbol: -P 1	Melting point = 473–472 K
*a* = 7.9679 (15) Å	Mo *K*α radiation, λ = 0.71073 Å
*b* = 9.4042 (19) Å	Cell parameters from 3163 reflections
*c* = 14.166 (3) Å	θ = 3.3–27.5°
α = 85.421 (6)°	µ = 0.44 mm^−^^1^
β = 76.727 (5)°	*T* = 103 K
γ = 86.002 (6)°	Block, yellow
*V* = 1028.4 (3) Å^3^	0.53 × 0.50 × 0.40 mm

### Data collection

**Table:**

Rigaku AFC10/Saturn724+ diffractometer	4626 independent reflections
Radiation source: fine-focus sealed tube	3906 reflections with *I* > 2σ(*I*)
graphite	*R*_int_ = 0.021
Detector resolution: 28.5714 pixels mm^-1^	θ_max_ = 27.5°, θ_min_ = 3.3°
phi and ω scans	*h* = −10→10
Absorption correction: multi-scan (*CrystalClear*; Rigaku/MSC, 2008)	*k* = −12→12
*T*_min_ = 0.801, *T*_max_ = 0.844	*l* = −16→18
9828 measured reflections	

### Refinement

**Table:**

Refinement on *F*^2^	Primary atom site location: structure-invariant direct methods
Least-squares matrix: full	Secondary atom site location: difference Fourier map
*R*[*F*^2^ > 2σ(*F*^2^)] = 0.033	Hydrogen site location: inferred from neighbouring sites
*wR*(*F*^2^) = 0.091	H-atom parameters constrained
*S* = 1.05	*w* = 1/[σ^2^(*F*_o_^2^) + (0.0486*P*)^2^ + 0.1912*P*] where *P* = (*F*_o_^2^ + 2*F*_c_^2^)/3
4626 reflections	(Δ/σ)_max_ = 0.002
275 parameters	Δρ_max_ = 0.37 e Å^−^^3^
0 restraints	Δρ_min_ = −0.25 e Å^−^^3^

### Special details

**Table:**

Geometry. All e.s.d.'s (except the e.s.d. in the dihedral angle between two l.s. planes) are estimated using the full covariance matrix. The cell e.s.d.'s are taken into account individually in the estimation of e.s.d.'s in distances, angles and torsion angles; correlations between e.s.d.'s in cell parameters are only used when they are defined by crystal symmetry. An approximate (isotropic) treatment of cell e.s.d.'s is used for estimating e.s.d.'s involving l.s. planes.
Refinement. Refinement of *F*^2^ against ALL reflections. The weighted *R*-factor *wR* and goodness of fit *S* are based on *F*^2^, conventional *R*-factors *R* are based on *F*, with *F* set to zero for negative *F*^2^. The threshold expression of *F*^2^ > σ(*F*^2^) is used only for calculating *R*-factors(gt) *etc*. and is not relevant to the choice of reflections for refinement. *R*-factors based on *F*^2^ are statistically about twice as large as those based on *F*, and *R*- factors based on ALL data will be even larger.

### Fractional atomic coordinates and isotropic or equivalent isotropic displacement parameters (Å^2^)

**Table:**

	*x*	*y*	*z*	*U*_iso_*/*U*_eq_	
Cl1	0.65348 (6)	0.39036 (4)	1.06940 (3)	0.02494 (11)	
Cl2	1.02033 (6)	0.23777 (5)	0.99130 (3)	0.03270 (13)	
S1	0.46927 (5)	0.38651 (4)	0.73411 (3)	0.01502 (10)	
N1	0.42851 (17)	0.12266 (14)	0.82588 (10)	0.0177 (3)	
N2	0.24614 (17)	0.20416 (14)	0.72255 (10)	0.0174 (3)	
N3	0.40431 (17)	0.83042 (14)	0.50231 (10)	0.0166 (3)	
N4	0.46548 (16)	0.64873 (13)	0.62050 (9)	0.0152 (3)	
C1	0.5466 (2)	0.24613 (16)	0.93887 (11)	0.0167 (3)	
H1	0.4370	0.2936	0.9610	0.020*	
C2	0.6847 (2)	0.27394 (16)	0.97755 (11)	0.0172 (3)	
C3	0.8457 (2)	0.20465 (18)	0.94480 (12)	0.0199 (3)	
C4	0.8656 (2)	0.1063 (2)	0.87532 (13)	0.0260 (4)	
H4	0.9749	0.0581	0.8535	0.031*	
C5	0.7274 (2)	0.07723 (19)	0.83720 (12)	0.0227 (4)	
H5	0.7415	0.0083	0.7901	0.027*	
C6	0.5687 (2)	0.14890 (16)	0.86789 (11)	0.0158 (3)	
C7	0.37223 (19)	0.22404 (16)	0.76455 (11)	0.0150 (3)	
C8	0.2218 (2)	0.32085 (16)	0.66094 (11)	0.0161 (3)	
C9	0.3305 (2)	0.42957 (16)	0.65541 (11)	0.0153 (3)	
C10	0.3515 (2)	0.56427 (16)	0.59808 (11)	0.0144 (3)	
C11	0.2660 (2)	0.60746 (16)	0.52437 (11)	0.0173 (3)	
H11	0.1906	0.5473	0.5052	0.021*	
C12	0.2970 (2)	0.74247 (17)	0.48071 (12)	0.0180 (3)	
H12	0.2372	0.7750	0.4316	0.022*	
C13	0.4895 (2)	0.77493 (16)	0.57055 (11)	0.0150 (3)	
C14	0.7437 (2)	0.82794 (16)	0.63641 (11)	0.0155 (3)	
C15	0.8818 (2)	0.91812 (16)	0.61470 (11)	0.0162 (3)	
H15	0.8825	0.9958	0.5675	0.019*	
C16	1.0172 (2)	0.89571 (16)	0.66104 (12)	0.0180 (3)	
H16	1.1084	0.9595	0.6468	0.022*	
C17	1.0204 (2)	0.78011 (17)	0.72849 (12)	0.0176 (3)	
H17	1.1144	0.7642	0.7595	0.021*	
C18	0.8856 (2)	0.68802 (17)	0.75029 (11)	0.0173 (3)	
C19	0.7475 (2)	0.71314 (16)	0.70470 (11)	0.0179 (3)	
H19	0.6546	0.6510	0.7205	0.021*	
C20	0.3733 (2)	−0.02305 (17)	0.82886 (13)	0.0224 (4)	
H20A	0.2555	−0.0291	0.8686	0.027*	
H20B	0.4515	−0.0895	0.8573	0.027*	
H20C	0.3759	−0.0482	0.7627	0.027*	
C21	0.0762 (2)	0.31764 (18)	0.61070 (13)	0.0248 (4)	
H21A	0.0086	0.4089	0.6166	0.030*	
H21B	0.0020	0.2399	0.6408	0.030*	
H21C	0.1229	0.3020	0.5418	0.030*	
C22	0.8862 (2)	0.56045 (18)	0.82177 (13)	0.0243 (4)	
H22A	0.8018	0.5787	0.8823	0.029*	
H22B	1.0015	0.5437	0.8352	0.029*	
H22C	0.8558	0.4761	0.7942	0.029*	
N5	0.61031 (17)	0.86089 (14)	0.58696 (10)	0.0185 (3)	
H5N	0.6034	0.9498	0.5630	0.032 (6)*	

### Atomic displacement parameters (Å^2^)

**Table:**

	*U*^11^	*U*^22^	*U*^33^	*U*^12^	*U*^13^	*U*^23^
Cl1	0.0352 (2)	0.0191 (2)	0.0227 (2)	0.00067 (16)	−0.00980 (17)	−0.00635 (15)
Cl2	0.0238 (2)	0.0468 (3)	0.0331 (3)	−0.00328 (19)	−0.01516 (19)	−0.0099 (2)
S1	0.01644 (19)	0.01321 (18)	0.0177 (2)	−0.00395 (13)	−0.00857 (15)	0.00174 (14)
N1	0.0186 (7)	0.0134 (6)	0.0232 (7)	−0.0041 (5)	−0.0095 (6)	0.0033 (5)
N2	0.0165 (7)	0.0164 (7)	0.0209 (7)	−0.0034 (5)	−0.0075 (5)	0.0005 (5)
N3	0.0178 (7)	0.0153 (6)	0.0177 (7)	−0.0006 (5)	−0.0067 (5)	0.0004 (5)
N4	0.0166 (6)	0.0143 (6)	0.0163 (7)	−0.0020 (5)	−0.0068 (5)	−0.0005 (5)
C1	0.0175 (8)	0.0151 (7)	0.0167 (8)	0.0007 (6)	−0.0040 (6)	0.0021 (6)
C2	0.0249 (8)	0.0130 (7)	0.0140 (8)	−0.0015 (6)	−0.0057 (6)	0.0016 (6)
C3	0.0174 (8)	0.0255 (8)	0.0182 (8)	−0.0026 (6)	−0.0070 (6)	−0.0004 (6)
C4	0.0165 (8)	0.0362 (10)	0.0261 (10)	0.0036 (7)	−0.0055 (7)	−0.0100 (7)
C5	0.0197 (8)	0.0295 (9)	0.0201 (9)	0.0011 (7)	−0.0048 (7)	−0.0095 (7)
C6	0.0166 (7)	0.0159 (7)	0.0148 (8)	−0.0027 (6)	−0.0048 (6)	0.0043 (6)
C7	0.0149 (7)	0.0138 (7)	0.0161 (8)	−0.0026 (6)	−0.0028 (6)	−0.0007 (6)
C8	0.0158 (7)	0.0153 (7)	0.0186 (8)	−0.0011 (6)	−0.0066 (6)	−0.0016 (6)
C9	0.0155 (7)	0.0155 (7)	0.0167 (8)	−0.0001 (6)	−0.0073 (6)	−0.0018 (6)
C10	0.0160 (7)	0.0128 (7)	0.0156 (8)	0.0001 (5)	−0.0056 (6)	−0.0025 (6)
C11	0.0184 (8)	0.0172 (8)	0.0187 (8)	−0.0016 (6)	−0.0090 (6)	−0.0019 (6)
C12	0.0199 (8)	0.0181 (8)	0.0172 (8)	0.0020 (6)	−0.0078 (6)	−0.0004 (6)
C13	0.0169 (7)	0.0152 (7)	0.0129 (7)	−0.0004 (6)	−0.0034 (6)	−0.0011 (6)
C14	0.0164 (7)	0.0161 (7)	0.0145 (8)	−0.0023 (6)	−0.0042 (6)	−0.0019 (6)
C15	0.0184 (8)	0.0136 (7)	0.0151 (8)	−0.0020 (6)	−0.0008 (6)	−0.0001 (6)
C16	0.0145 (7)	0.0177 (8)	0.0210 (8)	−0.0034 (6)	−0.0005 (6)	−0.0042 (6)
C17	0.0156 (8)	0.0205 (8)	0.0180 (8)	−0.0001 (6)	−0.0056 (6)	−0.0050 (6)
C18	0.0210 (8)	0.0169 (7)	0.0152 (8)	−0.0014 (6)	−0.0062 (6)	−0.0014 (6)
C19	0.0208 (8)	0.0168 (8)	0.0179 (8)	−0.0068 (6)	−0.0072 (6)	0.0007 (6)
C20	0.0230 (9)	0.0149 (8)	0.0304 (10)	−0.0057 (6)	−0.0090 (7)	0.0055 (7)
C21	0.0248 (9)	0.0227 (9)	0.0321 (10)	−0.0076 (7)	−0.0168 (7)	0.0028 (7)
C22	0.0303 (9)	0.0217 (9)	0.0249 (9)	−0.0067 (7)	−0.0149 (7)	0.0051 (7)
N5	0.0228 (7)	0.0146 (7)	0.0203 (7)	−0.0053 (5)	−0.0101 (6)	0.0051 (5)

### Geometric parameters (Å, °)

**Table:**

Cl1—C2	1.7304 (17)	C11—C12	1.380 (2)
Cl2—C3	1.7266 (17)	C11—H11	0.9500
S1—C7	1.7392 (15)	C12—H12	0.9500
S1—C9	1.7492 (15)	C13—N5	1.3694 (19)
N1—C7	1.361 (2)	C14—C19	1.394 (2)
N1—C6	1.424 (2)	C14—C15	1.399 (2)
N1—C20	1.4626 (19)	C14—N5	1.407 (2)
N2—C7	1.310 (2)	C15—C16	1.382 (2)
N2—C8	1.3778 (19)	C15—H15	0.9500
N3—C12	1.330 (2)	C16—C17	1.392 (2)
N3—C13	1.3561 (19)	C16—H16	0.9500
N4—C13	1.3346 (19)	C17—C18	1.391 (2)
N4—C10	1.3530 (19)	C17—H17	0.9500
C1—C2	1.386 (2)	C18—C19	1.396 (2)
C1—C6	1.386 (2)	C18—C22	1.507 (2)
C1—H1	0.9500	C19—H19	0.9500
C2—C3	1.394 (2)	C20—H20A	0.9800
C3—C4	1.380 (2)	C20—H20B	0.9800
C4—C5	1.384 (2)	C20—H20C	0.9800
C4—H4	0.9500	C21—H21A	0.9800
C5—C6	1.385 (2)	C21—H21B	0.9800
C5—H5	0.9500	C21—H21C	0.9800
C8—C9	1.372 (2)	C22—H22A	0.9800
C8—C21	1.497 (2)	C22—H22B	0.9800
C9—C10	1.447 (2)	C22—H22C	0.9800
C10—C11	1.392 (2)	N5—H5N	0.8800
			
C7—S1—C9	88.31 (7)	N4—C13—N3	126.28 (14)
C7—N1—C6	120.09 (13)	N4—C13—N5	119.49 (14)
C7—N1—C20	118.53 (13)	N3—C13—N5	114.23 (13)
C6—N1—C20	119.69 (12)	C19—C14—C15	118.38 (14)
C7—N2—C8	110.18 (13)	C19—C14—N5	124.83 (14)
C12—N3—C13	114.27 (13)	C15—C14—N5	116.80 (13)
C13—N4—C10	117.34 (13)	C16—C15—C14	120.89 (14)
C2—C1—C6	119.86 (15)	C16—C15—H15	119.6
C2—C1—H1	120.1	C14—C15—H15	119.6
C6—C1—H1	120.1	C15—C16—C17	120.25 (14)
C1—C2—C3	120.02 (15)	C15—C16—H16	119.9
C1—C2—Cl1	119.41 (12)	C17—C16—H16	119.9
C3—C2—Cl1	120.53 (13)	C18—C17—C16	119.82 (15)
C4—C3—C2	119.66 (15)	C18—C17—H17	120.1
C4—C3—Cl2	119.29 (13)	C16—C17—H17	120.1
C2—C3—Cl2	121.04 (13)	C17—C18—C19	119.58 (14)
C3—C4—C5	120.44 (16)	C17—C18—C22	121.23 (15)
C3—C4—H4	119.8	C19—C18—C22	119.20 (14)
C5—C4—H4	119.8	C14—C19—C18	121.06 (14)
C4—C5—C6	119.86 (16)	C14—C19—H19	119.5
C4—C5—H5	120.1	C18—C19—H19	119.5
C6—C5—H5	120.1	N1—C20—H20A	109.5
C5—C6—C1	120.11 (15)	N1—C20—H20B	109.5
C5—C6—N1	119.71 (15)	H20A—C20—H20B	109.5
C1—C6—N1	120.18 (14)	N1—C20—H20C	109.5
N2—C7—N1	122.48 (14)	H20A—C20—H20C	109.5
N2—C7—S1	115.90 (11)	H20B—C20—H20C	109.5
N1—C7—S1	121.59 (12)	C8—C21—H21A	109.5
C9—C8—N2	115.95 (14)	C8—C21—H21B	109.5
C9—C8—C21	127.26 (14)	H21A—C21—H21B	109.5
N2—C8—C21	116.74 (13)	C8—C21—H21C	109.5
C8—C9—C10	132.96 (14)	H21A—C21—H21C	109.5
C8—C9—S1	109.62 (11)	H21B—C21—H21C	109.5
C10—C9—S1	117.41 (11)	C18—C22—H22A	109.5
N4—C10—C11	120.73 (14)	C18—C22—H22B	109.5
N4—C10—C9	114.52 (13)	H22A—C22—H22B	109.5
C11—C10—C9	124.75 (14)	C18—C22—H22C	109.5
C12—C11—C10	116.32 (14)	H22A—C22—H22C	109.5
C12—C11—H11	121.8	H22B—C22—H22C	109.5
C10—C11—H11	121.8	C13—N5—C14	129.52 (13)
N3—C12—C11	124.83 (14)	C13—N5—H5N	115.2
N3—C12—H12	117.6	C14—N5—H5N	115.2
C11—C12—H12	117.6		
			
C6—C1—C2—C3	0.3 (2)	C7—S1—C9—C8	1.59 (12)
C6—C1—C2—Cl1	−177.54 (11)	C7—S1—C9—C10	−177.37 (13)
C1—C2—C3—C4	−1.4 (2)	C13—N4—C10—C11	1.2 (2)
Cl1—C2—C3—C4	176.42 (13)	C13—N4—C10—C9	−179.55 (13)
C1—C2—C3—Cl2	179.72 (12)	C8—C9—C10—N4	174.45 (17)
Cl1—C2—C3—Cl2	−2.50 (19)	S1—C9—C10—N4	−6.89 (19)
C2—C3—C4—C5	0.8 (3)	C8—C9—C10—C11	−6.3 (3)
Cl2—C3—C4—C5	179.69 (14)	S1—C9—C10—C11	172.32 (13)
C3—C4—C5—C6	1.0 (3)	N4—C10—C11—C12	−3.6 (2)
C4—C5—C6—C1	−2.1 (2)	C9—C10—C11—C12	177.22 (15)
C4—C5—C6—N1	177.66 (15)	C13—N3—C12—C11	2.2 (2)
C2—C1—C6—C5	1.5 (2)	C10—C11—C12—N3	1.8 (2)
C2—C1—C6—N1	−178.28 (13)	C10—N4—C13—N3	3.5 (2)
C7—N1—C6—C5	−108.63 (18)	C10—N4—C13—N5	−176.52 (14)
C20—N1—C6—C5	56.3 (2)	C12—N3—C13—N4	−5.1 (2)
C7—N1—C6—C1	71.1 (2)	C12—N3—C13—N5	174.89 (14)
C20—N1—C6—C1	−123.93 (16)	C19—C14—C15—C16	−1.2 (2)
C8—N2—C7—N1	−177.07 (14)	N5—C14—C15—C16	178.77 (14)
C8—N2—C7—S1	0.87 (18)	C14—C15—C16—C17	1.7 (2)
C6—N1—C7—N2	178.64 (14)	C15—C16—C17—C18	−0.9 (2)
C20—N1—C7—N2	13.5 (2)	C16—C17—C18—C19	−0.5 (2)
C6—N1—C7—S1	0.8 (2)	C16—C17—C18—C22	179.02 (15)
C20—N1—C7—S1	−164.32 (12)	C15—C14—C19—C18	−0.2 (2)
C9—S1—C7—N2	−1.46 (13)	N5—C14—C19—C18	179.85 (15)
C9—S1—C7—N1	176.49 (14)	C17—C18—C19—C14	1.0 (2)
C7—N2—C8—C9	0.5 (2)	C22—C18—C19—C14	−178.47 (16)
C7—N2—C8—C21	−177.22 (14)	N4—C13—N5—C14	14.1 (2)
N2—C8—C9—C10	177.21 (16)	N3—C13—N5—C14	−165.88 (15)
C21—C8—C9—C10	−5.4 (3)	C19—C14—N5—C13	−21.4 (3)
N2—C8—C9—S1	−1.52 (18)	C15—C14—N5—C13	158.68 (16)
C21—C8—C9—S1	175.88 (14)		

### Hydrogen-bond geometry (Å, °)

**Table:**

*D*—H···*A*	*D*—H	H···*A*	*D*···*A*	*D*—H···*A*
N5—H5N···N3^i^	0.88	2.20	3.078 (2)	177

Symmetry codes: (i) −*x*+1, −*y*+2, −*z*+1.
